# *Nerium oleander* Distillate Improves Fat and Glucose Metabolism in High-Fat Diet-Fed Streptozotocin-Induced Diabetic Rats

**DOI:** 10.1155/2012/947187

**Published:** 2012-12-02

**Authors:** Ahmet Levent Bas, Sule Demirci, Nuray Yazihan, Kamil Uney, Ezgi Ermis Kaya

**Affiliations:** ^1^Pharmacology and Toxicology Department, Faculty of Veterinary Medicine, Selcuk University, 42070 Konya, Turkey; ^2^Physiology Department, Faculty of Veterinary Medicine, Mehmet Akif Ersoy University, 15030 Burdur, Turkey; ^3^Pathophysiology Department, Faculty of Medicine, Ankara University, 06100 Ankara, Turkey; ^4^Molecular Biology Research and Development Unit, Faculty of Medicine, Ankara University, 06100 Ankara, Turkey

## Abstract

Diabetes was induced by intraperitoneal injection of streptozotocin (35 mg/kg bw) in all rats of five groups after being fed for 2 weeks high-fat diet. Type 2 diabetic *Nerium-oleander-* (NO-) administered groups received the NO distillate at a dose of 3.75, 37.5, and 375 **μ**g/0.5 mL of distilled water (NO-0.1, NO-1, NO-10, resp.); positive control group had 0.6 mg glibenclamide/kg bw/d by gavage daily for 12 weeks. Type 2 diabetic negative control group had no treatment. NO distillate administration reduced fasting blood glucose, HbA1c, insulin resistance, total cholesterol, low density lipoprotein, atherogenic index, triglyceride-HDL ratio, insulin, and leptin levels. Improved beta cell function and HDL concentration were observed by NO usage. HDL percentage in total cholesterol of all NO groups was similar to healthy control. NO-10 distillate enhanced mRNA expressions of peroxisome proliferator-activated-receptor- (PPAR-) **α**, **β**, and **γ** in adipose tissue and PPAR-**α**–**γ** in liver. The findings from both *in vivo* and *in vitro* studies suggest that the considerable beneficial effect of NO distillate administration at a dose of 375 **μ**g/0.5 mL of distilled water may offer new approaches to treatment strategies that target both fat and glucose metabolism in type 2 diabetes.

## 1. Introduction

The cluster of pathologies known as metabolic syndrome,including obesity, insulin resistance (IR),type 2 diabetes, and cardiovascular disease (CVD), has become one of the most serious threats to human health. The dramatic increase in the incidence of obesity in most parts of the world has contributed to the emergence of this disease cluster, particularly insulin resistance and type 2 diabetes [1].

IR is associated with a number of diseases including obesity, metabolic syndrome, type 2 diabetes, lipodystrophies, polycystic ovary syndrome, and chronic infection. The overall prevalence of IR is reported to be 10–25% [2]. The main characteristics of IR are disinhibited lipolysis in adipose tissue, impaired uptake of glucose by muscle, and disinhibited gluconeogenesis [2].

IR most often precedes the onset of overt type 2 diabetes and is compensated initially by hyperinsulinemia [3]. But this chronic secretion of large amounts of insulin to overcome tissue insensitivity can itself finally lead to beta cell failure and occurrence of hyperglycemia [4].

In IR, visceral adipose tissue is resistant to the antilipolytic effect of insulin and consequently releases excessive amounts of FFA. A major contributor to the development of IR is an overabundance of circulating fatty acids. Insulin-resistant people with obesity and/or type 2 diabetes have been identified a defect in mitochondrial oxidative phosphorylation that relates to the accumulation of triglycerides and related lipid molecules in muscle [5].

In type 2 diabetes, elevated blood glucose levels are clearly an important secondary cause of dyslipidemia in susceptible patients, and poor control of glycaemia can sometimes result in profound dyslipidaemia, including hypertriglyceridaemia and low high density lipoprotein (HDL) blood levels [6]. Sustained hyperglycemia in type 2 diabetes induces macrovascular and microvascular complications. In a recent report of World Health Organization and International Diabetes Federation it was found that about 80% diabetic morbidity and mortality is caused by diabetic cardiomyopathy which is closely related with diabetic dyslipidemia [7].

The low density lipoprotein (LDL) blood level in a patient with diabetes may be somewhat misleading; a patient with diabetes may have an increased proportion of small dense LDL particles and an increase in atherogenic risk, compared with a nondiabetic patient with similar LDL blood level. Moreover, patients with small, dense LDL will also typically have lower HDL and elevated triglyceride blood levels, which may further increase atherosclerosis risk [6]. 

Plant derivatives with purported hypoglycemic properties have been used in folk medicine and traditional healing systems around the world [8]. Many modern pharmaceuticals used in conventional medicine today also have natural plant origins. Among them, metformin was derived from the flowering plant *Galega officinalis* (Goat's Rue or French Lilac), which was a common traditional remedy for diabetes [9].

 Oleander (*Nerium oleander, *NO) of the dogbane (Apocynaceae) family grows along the whole Mediterranean coast starting in southern Portugal in the west, in Syria, and in streambeds of the Western and Southern Anatolia. Central nervous system depressant activity [10] and dose-dependent cardiotonic effect [11] of NO were exhibited in studies. NO has also been used in the treatment of cancer [12]. Ishikawa et al. [13] were reported that postprandial rise in blood glucose when maltose and sucrose were loaded in nondiabetic healthy rats was reduced by hot water extract of *Nerium indicum* leaves. Recently ligands for the peroxisome proliferator-activated receptors (PPARs) which play a key role in glucose and lipid metabolism are defined as new insulin sensitizing drugs and hypolipidemic fibrates. 

In the present study, we investigated the effects of NO distillate on hyperglycemia and dyslipidemia and its activities on liver and adipose tissue PPARs in type 2 diabetic rats.

## 2. Materials and Methods 

### 2.1. Obtaining Lyophilized NO and Dosage Administration

NO plant was collected among new shoots in March–September period from Mediterranean region of Turkey, identified, and authenticated at the Department of Biology. Firstly collected plant was washed, fresh shoots were chopped, and adequate distilled water was added. The mixture was heated in heat resistant container. After the liquid started to evaporate, container lid was covered, and vapor was separated to other clean glass containers by causing it to come in contact with a surface cooled with cold water. NO distillate was lyophilized in small glass bottle (20 mL) by using lyophilizator. Lyophilized NO distillates were dissolved at concentrations of 7.5, 75, and 750 *μ*g/mL in distilled water. 

### 2.2. Animals

Eighty male Sprague Dawley rats (10–12 weeks) were allocated to metabolic cages individually in an automatic ambient humidity (50 ± 5%), temperature (22 ± 2°C), and light-dark (12 : 12) controlled room. Animals were obtained from the Experimental Research Center of Akdeniz University, Faculty of Medicine, Antalya, Turkey and divided into eight groups. Each healthy group had six rats; other experimental groups had ten rats. Commercially available rat normal pellet diet and water were given ad libitum to all animals prior to dietary manipulation. The experimental protocol was approved by the Ethics Committee in Animal Experimentation of Selcuk University, Turkey.

### 2.3. Induction of Diabetes

After 2 weeks feeding of high fat diet, diabetes was induced in fasted animals by a single intra-peritoneal injection of streptozotocin (STZ) (35 mg/kg bw) dissolved in citrate buffer (pH 4.5) [14] when only buffer was received by control groups. In one week after STZ injection, rats with 16.65 mmol/L (300 mg/dL) nonfasting blood glucose level were considered to be type 2 diabetic. Chemicals were obtained from Sigma (MO, USA) otherwise stated.

### 2.4. Diets, Feeding, and Experimental Design

Healthy control (C) and healthy control administered the highest level of NO (CNO-10) group had normal pellet diet although high fat group (HF) and other all type 2 diabetic groups had high fat diet which 58% of the metabolic energy is provided from animal fat. Nutritional substances of normal pellet diet were dry matter 89%, crude protein 21%, metabolic energy 2850 kcal/kg, crude fiber 5%, methionine and cystein 0.75%, calcium 1.0–2.0%, phosphor 0.5–1.0%, and sodium 0.5% (Optima Feeds, Turkey). The details of high fat diet composition were given in Table 1. 

After induction of diabetes, animals were randomly allocated to five groups in which one of them did not had any treatment (D); other groups had active substances once a day by gavage for 12 weeks of the treatment period. 

The experimental groups according to diets and administrations applied to animals were represented in Table 2. 

### 2.5. Sampling and Analytical Methods

Body weight change of each animal was noted weekly. Although fasting blood samples were taken from tail vein of all rats at 15 day intervals through the experiment, the results in Tables 3 and 4 represent only the last sampling. About 0.5 mL whole blood from each animal was collected in EDTA tubes for analysis HbA1c at the end of the experiment. Remaining blood was collected and centrifuged to separate serum. Serum samples were analyzed immediately for fasting blood glucose (FBG), total cholesterol (total-C), HDL, LDL, triglyceride, alkaline phosphatase (ALP), aspartate transaminase (AST), and alanine transaminase (ALT) by using commercially available colorimetric diagnostic kits (IL Test, Instrumentation Laboratory, Milano, Italy) by autoanalyzer (ILab, 300, Milano, Italy). After sampling the animals in all test groups was euthanized under general anesthesia with thiopental sodium (50 mg/kg bw) subsequent to the final sampling time. Liver and white adipose tissue samples from subcutan adipose tissue were taken and kept under required conditions.

IR was evaluated by the homeostasis model assessment and expressed in HOMA-IR. Data for HOMA-IR (HOMA-IR: fasting insulin (*μ*U/mL) × fasting glucose (mmol/L)/22.5), beta cell functions as HOMA-*β* (HOMA-*β*(%): (20 × fasting insulin (*μ*U/mL)/{fasting glucose (mmol/L) *‒* 3.5}) [15], atherogenic index (AI: ([Total-C] − [HDL])/[HDL]) [16], HDL % in total-C, and triglyceride to HDL ratio of the all studied groups were calculated.

Serum insulin (DRG, Millipore, MA, USA) and leptin (R&D, MN, USA) levels were analyzed by ELISA according to kit procedures.

### 2.6. Evaluation of PPAR-*α*, -*β*, -*γ* mRNA Expressions

Total RNA is extracted, and oligo-dT primed first-strand cDNA is synthesized. A reverse transcription polymerase chain reaction (RT-PCR) is performed using a thermal cycler system, specific primers for PPAR-*α*, PPAR-*γ*, and *β*-actin (Roche Diagnostics, Rotkreuz, Switzerland) are used. *β*-Actin is used as an internal control. 

### 2.7. Statistical Analysis

Results were presented as mean ± SEM. *In vivo* data analyses were performed one-way ANOVA followed by a multiple comparison test (postdoc Duncan's test) using SPSS 17.0 (SPSS, Chicago, USA). Differences were considered significant at *P* less than 0.05. Also Kruskall Wallis and Mann Whitney-*U* test (use these when the data is not normally distributed) were used to determine statistically differences of *in vitro* data between groups. The correlation statistics were evaluated using Pearson correlation coefficient.

## 3. Results 

Table 3 shows values of body weight, FBG, HbA1c, total-C, HDL, LDL, and triglyceride, AI, HDL% in total-C, triglyceride to HDL ratio of the all studied groups. Data for insulin, leptin, HOMA-IR, HOMA-*β*, ALP, AST, and ALT were presented in Table 4.

No gastrointestinal disorders were observed during or after NO treatment. Although being diabetic, the considerable elevation in body weight of NO treated groups was estimated compared to D and G (*P* < 0.0001). CNO-10 had numerically lower body weight than C (Table 3). FBG levels were significantly decreased by using NO when compared to D and G (*P* < 0.0001). In parallel with the improvement of FBG, there was a significant reduction in HbA1c in animals administered NO. As shown in Table 3, all other diabetic groups were hyperglycemic and had significantly higher HbA1c than healthy groups and diabetic NO groups (*P* < 0.0001). Although having high fat diet, NO-1 and NO-10 displayed similar total-C concentrations compared to C (*P* > 0.05, Table 3). Total-C was numerically lower in CNO-10 and higher in HF than C. 

The increased values of HDL were found in NO-0.1 and NO-10 compared to C (*P* < 0.0001, Table 3). The reducing effect of all NO regimens and G on LDL concentration was noticeable and the values were similar to healthy groups (*P* > 0.05). LDL levels in type 2 diabetic NO-1 and NO-10 groups were significantly lower than D (*P* < 0.05). HDL percentage in total-C of NO-0.1 and NO-1 groups was similar to C (*P* > 0.05) and the highest HDL percentage was estimated in NO-10 among healthy and diabetic groups except CNO-10. The lowest triglyceride concentration was found in CNO-10 among the healthy rats. 

The similarity in terms of AI of NO groups to healthy groups was noticeable (*P* > 0.05, Table 3). The reducing effect of NO-10 on AI was significant when compared to D, G, NO-0.1, and NO-1 (*P* < 0.001). 

There were significant reductions in triglyceride-HDL ratio of all NO regimens compared to D (*P* < 0.0001, Table 3). These reducing effects of NO on the ratio were noticeable and the results were similar to C (*P* > 0.05). Triglyceride-HDL ratio was numerically lower in CNO-10 and was higher in HF than C. Similar results in G and D were noted in terms of triglyceride-HDL ratio. 

When we assessed insulin levels, the antihyperglycemic effect of NO was seen on data that NO significantly decreased insulin concentration compared to D (*P* < 0.0001, Table 4). Although insulin levels in all NO groups were numerically higher than other healthy control groups, the results were statistically similar with those groups (*P* > 0.05).Insulin level was decreased by G and data were not different with all NO groups and healthy groups (*P* > 0.05). There was 26.72% fall in insulin level of CNO-10 compared to C.

Leptin levels of all NO groups were not significantly different compared to healthy control groups (*P* > 0.05). Almost twofold increases were estimated in other diabetic groups when compared to all NO treated groups (*P* < 0.01, Table 4)

Calculated HOMA-IR in NO groups and in G was significantly lower than D (*P* < 0.0001, Table 4). Insulin sensitivity has been improved by NO treatment. Also numerically the lowest insulin resistance was found in CNO-10.

HOMA-*β* impaired dramatically in groups D and G. Improved beta cell function was observed in all NO groups and was similar to healthy group's values (*P* > 0.05, Table 4). 

The highest ALP activity was found in D (*P* < 0.0001, Table 4). All NO treatment dosages decreased ALP activity when compared to D (*P* < 0.0001). The ALP activity of G was similar to all NO regimens (*P* > 0.05). NO-10 administration reduced AST activity compared to D (*P* < 0.05). The elevated ALT activities, compared with C, were observed in D and G (*P* < 0.05, Table 4).

**Figure 1:**
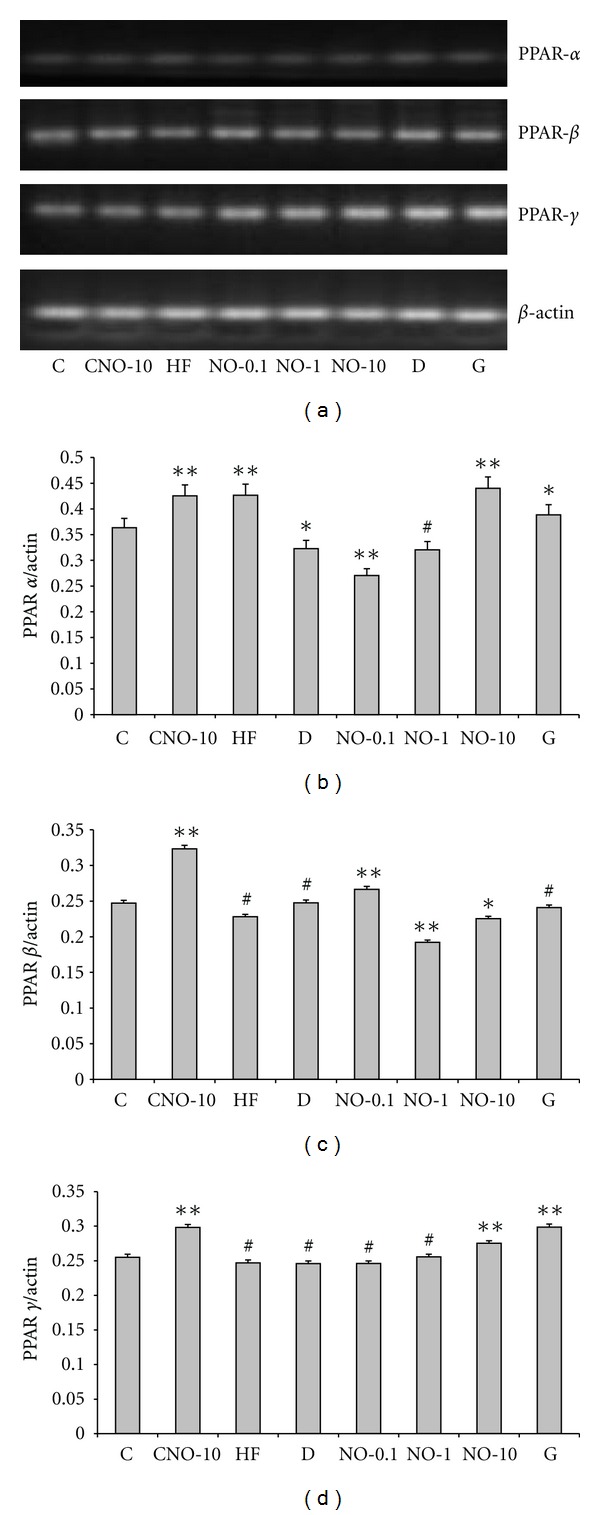
(a) PPAR-*α*, -*β*, -*γ* mRNA expressions in rat liver tissue. (b) The ratio of PPAR-*α* to *β*-actin mRNA expressions in liver tissue. (statistics are given as groups compared to control, *P < 0.05, **P < 0.001, ^#^ *P* > 0.05). (c) The ratio of PPAR-*β* to *β*-actin mRNA expressions in liver tissue. (Statistics are given as groups compared to control, *P < 0.05, **P < 0.001, ^#^ *P* > 0.05) (d) The ratio of PPAR-*γ* to *β*-actin mRNA expressions in liver tissue. (statistics are given as groups compared to control, *P < 0.05, **P < 0.001, ^#^ *P* > 0.05) C, healthy control, normal diet; CNO-10, healthy control, normal diet, and NO distillate at a dose of 375 *μ*g/0.5 mL of distilled water/d; HF, healthy control high fat diet; NO-0.1, type 2 diabetic, high fat diet, and NO distillate at a dose of 3.75 *μ*g/0.5 mL of distilled water/d; NO-1, type 2 diabetic, high fat diet, and NO distillate at a dose of 37.5 *μ*g/0.5 mL of distilled water/d; NO-10, type 2 diabetic, high fat diet, and NO distillate at a dose of 375 *μ*g/0.5 mL of distilled water/d; D, type 2 diabetic, high fat diet; G, type 2 diabetic, high fat diet, and glibenclamide at a dose of 0.6 mg/kg bw/0.5 mL of distilled water/d.

**Figure 2:**
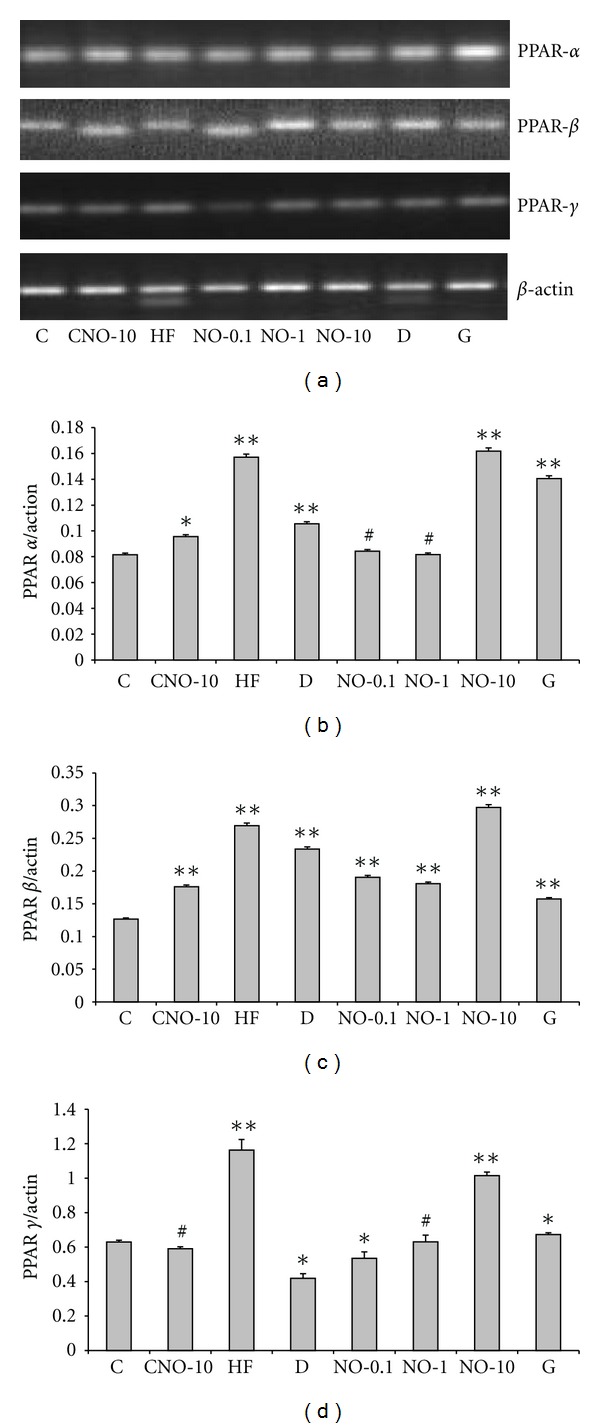
(a) PPAR-*α*, -*β*, -*γ* mRNA expressions in rat adipose tissue. (b) The ratio of PPAR-*α* to *β*-actin mRNA expressions in adipose tissue. (statistics are given as groups compared to control, *P < 0.05, **P < 0.001, ^#^ *P* > 0.05). (c) The ratio of PPAR-*β* to *β*-actin mRNA expressions in adipose tissue. (statistics are given as groups compared to control, *P < 0.05, **P < 0.001, ^#^ *P* > 0.05) (d) The ratio of PPAR-*γ* to *β*-actin mRNA expressions in adipose tissue. (Statistics are given as groups compared to control, *P < 0.05, **P < 0.001, ^#^ *P* > 0.05) C, healthy control, normal diet; CNO-10, healthy control, normal diet, and NO distillate at a dose of 375 *μ*g/0.5 mL of distilled water/d; HF, healthy control high fat diet; NO-0.1, type 2 diabetic, high fat diet, and NO distillate at a dose of 3.75 *μ*g/0.5 mL of distilled water/d; NO-1, Type 2 diabetic, high fat diet, and NO distillate at a dose of 37.5 *μ*g/0.5 mL of distilled water/d; NO-10, type 2 diabetic, high fat diet, and NO distillate at a dose of 375 *μ*g/0.5 mL of distilled water/d; D, type 2 diabetic, high fat diet; G, Type 2 diabetic, high fat diet, and glibenclamide at a dose of 0.6 mg/kg bw/0.5 mL of distilled water/d.

The primer sequences, PCR protocol, and product sizes are presented in Table 5. Agarose gel electrophoresis of PCR products are shown in Figures 1(a) and 2(a). The densities of each band evaluated by Diana V1.6 and Aida 2.4.3 analysis programs (Raytest Imaging system, Germany) and the ratio of liver and adipose tissues PPAR-*α*, -*β*, -*γ* mRNA expressions to *β*-actin are given in Figures 1(b), 1(c), 1(d) , 2(b), 2(c), and 2(d), respectively.

In liver, PPAR-*α* mRNA expression decreased in D compared to C (*P* < 0.05), whereas its expression increased in NO-10 treated group (*P* < 0.01). PPAR-*β* mRNA expression increased in NO-0.1 (*P* < 0.001) treated group compared to D. PPAR-*γ* mRNA expression increased in NO treated groups as a dose dependent manner but the increase in NO-10 group is not significant compared to C (*P* > 0.05).

In adipose tissue, PPAR-*α* and -*β* mRNA expression increased in NO-10 group compared to C and diabetic rats (*P* < 0.01). The increase in PPAR-*γ* mRNA expression is more prominent in NO-10 treated group compared to C and diabetic group (*P* < 0.001). During the experiment, administration of combined drug and insulin was not in question.

## 4. Discussion

Studies, most notably the DCCT, have defined quantitatively the relationship between glycated Hb and average glycemia [17]. The increase in vascular disease in patients with diabetes is thought to be due to the deleterious effects of metabolic abnormalities, such as hyperglycemia, insulin resistance, dyslipidemia, and advanced glycation end products [18, 19]. In diabetes, HbA1c levels predict the risk of microvascular complications [20] and glycemic control to a HbA1c of less than 7% will reduce microvascular complications and could decrease risk for macrovascular disease as well [21].

The most important result in terms of the FBG is that NO administered groups exhibited a level which was close to the data from C and that this condition was confirmed with the HbA1c levels obtained from the above-mentioned groups.

NO treatment significantly reduced HbA1c levels that were similar to healthy groups when glibenclamide had no significant effect over that. NO treatment reduced HbA1c 15.97, 15.94, and 19.54% in NO-0.1, NO-1, and NO-10, respectively, when compared to D. All NO regimens also decreased FBG compared to other diabetic groups at the end of trial. Therefore, NO may be beneficial to reduce microvascular and macrovascular risk of type 2 diabetes.

HOMA-IR is an independent predictor of CVD in type 2 diabetes and the improvement of IR might have beneficial effects not only on glucose control but also on CVD in patients with type 2 diabetes mellitus [22].

NO administration resulted in significant lowering of both insulin level and HOMA-IR compared to D as observed in G. Improved beta cell function (HOMA-*β*) in all NO dosages was established but the improvement was noticeable in NO-0.1.

HOMA-*β* was numerically higher in CNO-10 than C. The evaluated data suggest that NO ameliorates glycemic control by insulin secretagogue and sensitizing effects. 

Dyslipidemia, as associated with diabetic metabolism and the metabolic syndrome, is characterized by a so-called proatherogenic blood lipid profile, comprising low levels of HDLs, increased LDLs, and serum triglycerides associated with VLDLs [23]. In fact, hypertriglyceridemia is considered to represent an important risk factor for atherosclerosis and subsequent cardiovascular complications in type 2 diabetic patients [24].

The atherogenic index has recently been proposed as a marker of plasma atherogenicity because it is increased in people at higher risk for coronary heart disease and is inversely correlated with LDL particle size [25]. The increased risk of coronary heart disease in patients with the metabolic syndrome suggests that the insulin-resistant state is atherogenic without concomitant elevations in plasma glucose and glycosylated hemoglobin [26, 27]. The present data showed that all NO administration numerically decreased AI but the highest NO administration level significantly lowered atherogenicity in blood compared to other diabetic groups. 

A positive correlation between mean HOMA-IR and AI (*P* < 0.05, *r* = 0.71) and also between mean HbA1c and AI (*P* < 0.01, *r* = 0.86) suggests that improving blood glucose level and insulin receptor sensitivity may support to decline atherogenicity and cardiovascular complications in type 2 diabetes by using NO distillate. Moreover, the highly significant positive correlation between HOMA-IR and triglyceride levels (*P* < 0.0001, *r* = 0.74) and inclination of the reduction of triglyceride by NO administration indicates NO may have a good beneficial effect on glucose and lipid metabolism as inferred from HOMA-IR results.

The atherogenic link between high triglycerides and HDL is due to the higher plasma concentration of triglyceride-rich, VLDL that generates small, dense LDL during lipid exchange and lipolysis. These LDL particles accumulate in the circulation and form small, dense HDL particles, which undergo accelerated catabolism, thus closing the atherogenic circle [28, 29].

The significant similarity of C levels especially in total-C of NO-1 and NO-10 administration and LDL concentrations in all NO regimens of diabetics support lipid lowering effects of NO. 

Diabetic dyslipidemia includes an overall increase in atherogenic particles identifiable, by measuring apolipoprotein B, and a predominance of small dense LDL particles (phenotype B). A high ratio of triglycerides to HDL-cholesterol correlates with LDL phenotype B, small HDL particles, and IR [30–32] and found to be a powerful independent indicator of extensive coronary disease [33].

According to the above explanations, the ratio of triglycerides to HDL results in this paper indicates that NO treatment may prevent the extensive coronary disease due to decreasing atherogenic particles since the ratio found to be statistically very similar to the ratio of C. Also, in healthy rats, NO made reduction in triglycerides to HDL ratio.

High HDL levels in blood are considered to be “cardioprotective”, since the apo-A-containing HDL particles that help transport cholesterol to the liver from peripheral tissues,as well as away from macrophages associated with cholesterol deposits within the vascular wall. However, this cardioprotective effect may not be solely due to cholesterol transport. For example, HDL may have direct antioxidant and anti-inflammatory effects on the vessel wall [6].

The reduction in triglyceride levels together with the higher concentrations of HDL especially in NO-0.1 and NO-10 groups compared to C were remarkable effects of NO over dyslipidemia of type 2 diabetic rats. The ratio of serum HDL cholesterol to total-C used a marker of cardiovascular risk [34]. All NO treatment levels were associated with increase in HDL percentage in total-C but a highly significant arise was found in NO-10 compared to other diabetics although the lower percentages of HDL in total-C of diabetic groups were estimated especially in G and D. Numerically increase in HDL percent in total-C of CNO-10 compared to C also indicates the beneficial effect of NO over the lipid metabolism of healthy individuals.All these positive results in respect to lipid lowering effects of NO may indicate reducing risk factor for atherosclerosis and subsequent cardiovascular complications in type 2 diabetes. 

Leptin is a hormone secreted predominantly by adipose tissue and is a signal of sufficiency of energy. It decreases food intake and increases energy expenditure, thereby indirectly promoting insulin sensitivity. Leptin effects are mediated by its action on the hypothalamus and directly on target tissues (muscle, gonads, beta-cells, and liver). In normal conditions of weight maintenance, leptin concentrations are positively correlated with total body fat mass. In short-term food deprivation, serum leptin levels decrease and the opposite is true for short-term overfeeding. Soluble leptin receptors are thought to be important for transport to or over the blood–brain barrier (BBB), and it is the saturation of this transport or impairment of leptin receptor signal transduction that may be the cause of leptin resistance [2]. So that, the significant elevated levels of leptin in D and G, except NO treated ones, reflect leptin resistance and/or insulin resistant state. The effect of leptin on inducing insulin resistance was exhibited by positive correlation between mean HOMA-IR and leptin (*P* < 0.05, *r* = 0.76) in this research. 

NO positively affected leptin levels and was found to be similar to healthy control level. This remarkable result indicates that NO may help to compensate the energy metabolism by improving leptin and insulin levels, and HOMA-IR, HOMA-*β* in type 2 diabetes. 

Triglycerides are an important cause of leptin resistance as mediated by impaired transport across the BBB [35]. In this research, positive correlation was found between mean triglyceride and leptin levels (*P* < 0.05, *r* = 0.70). This data may indicate that high triglyceride levels resulted in high blood leptin levels in D and G groups.

A number of studies have reported that ALT, AST, and/or GGT levels independently predict incidents of type 2 diabetes, metabolic syndrome, and CVD [36]. In addition, these markers have been shown to be associated with indirect measures of insulin resistance including fasting insulin levels and HOMA-IR [37, 38] since ALT was associated with insulin resistance independently and an inexpensive way to improve the identification of subjects with insulin resistance [39]. 

The reduction of AST activity in NO-10 was noticeable when compared to D. ALT activity was significantly higher in D than C group but was similar in NO groups compared with C (*P* > 0.05). Also, mean ALT activity was positively correlated with HOMA-IR (*P* < 0.05, *r* = 0.80). 

Although ALP activities were not different between type 2 diabetic and nondiabetic patients [40], the increased ALP activity has been reported in ketotic and nonketotic diabetic rats primarily due to an increase in intestinal and bone/liver ALP isoenzyme [41]. All NO regimens exhibited the beneficial effect on reducing ALP activity in type 2 diabetic rats. The results of liver enzymes by using NO dosages show that NO had no detrimental effect on the liver function.

In rodents, adipose tissue PPAR-*γ* mRNA and protein levels are reduced after an overnight fast [42, 43] in STZ-induced diabetes [42], which is consistent with the stimulatory effect of insulin on PPAR-*γ* expression [44]. In addition, chronic feeding with high fat diets was shown to increase PPAR-*γ* expression in adipose tissue, whereas fasting stimulates especially liver PPAR-*α* expression in rodents [42, 43].

The overexpressions of PPAR-*β* and -*γ* in adipose tissue may indicate that the effect of NO-10 group dosage on reducing insulin resistance and indirectly reducing the risk of atherosclerosis is being through the storage of fatty acids to adipocytes [45] and regulating adipocyte differentiation [46].

Significantly increased PPAR-*α* expression in liver and adipose tissue of NO-10 group suggests that NO-10 dosage may have important effects on regulating fatty acid oxidation system, lipoprotein synthesis, and inflammatory responses [46].

Single agents that promote both PPAR-*α* and PPAR-*γ* agonism could theoretically offer significant benefits in improving dyslipidaemia and reducing hyperglycaemia and thus reduce these cardiovascular risk factors associated with type 2 diabetes and metabolic syndrome. In addition, such a therapy could reduce the underlying insulin resistance and help to break the cycle of altered glucose and lipid metabolism that promotes type 2 diabetes [6].

It has been considered that increasing fatty acid oxidation in liver by overexpression of liver PPAR-**α** [46] led not to use too much fat from adipose tissues for energy and also storage of fatty acids in adipocytes by overexpression of adipose tissue PPAR-**γ** [45] of NO treated animals improved insulin action. This progress prevented body weight loss of NO treated animals due to an antilipolitic effect of insulin when compared to uncontrolled diabetic ones. This effect of NO was statistically significant especially in NO-10 group. HOMA-IR and HOMA-*β* results also support this improvement.

Also the pulling down effect of NO-10 dosage on body weight might be associated with overexpressions of PPAR-*α* and -*β* in adipose tissue of healthy rats since overexpression of PPAR-*β* specifically in adipose tissue decreases fatty acid levels and protects against obesity [47]. Moreover PPAR-*α* and PPAR-*β* are both active participants in energy burning; PPAR-*α* plays a critical role in fatty acid oxidation and is thus responsible for energy expenditure; PPAR-*β* also enhances fatty acid catabolism and energy uncoupling in skeletal muscle and adipose tissue as well, as recently shown in the liver [46].

## 5. Conclusions

The considerable beneficial effects of especially NO-10 group treatment (NO distillate administration at a dose of 375 *μ*g/0.51 mL of distilled water/d) on glucose metabolism, insulin resistance, insulinotropic activity, leptin, dyslipidemia, liver enzymes, and PPARs may point out the insulin-like effect of NO and offer new approaches to treatment strategies that target both fat and glucose metabolism ultimately leadind to a reduction in both the chronic microvascular complications of type 2 diabetes and the risk of macrovascular events such as CVD. In addition, this concentration of NO may prevent and control elevated glucose and blood lipid levels of the remainder of the population.

## Figures and Tables

**Table 1:** High fat diet composition and nutritional substances.

Ingredients	%	Nutritional substances	%
Vegetable oil	3.00	DM	94.4
		CP	22.3
Animal fat (tallow)	37.00	ME, kcal/kg	5387
Corn, yellow	30.50	Ca	1.16
Casein, dried	20.00	P	0.62
Soya pulp, 48%	4.50	Na	0.21
Dicalcium phosphate	1.70	Met + Cys	0.94
Dl-methionine	0.20	Lysine	1.81
Lime stone	1.60	CF	0.89
Salt	0.50	EE	40.34
Vitamin-mineral	1.00	Linoleic acid	3.40

DM: dry matter; CP: crude protein, ME: metabolic energy: CF: crude fiber; EE: ether extract; Met + Cys: methionine and cystein.

**Table 2:** The implementation of experimental groups according to diets and administrations applied to animals.*

	Healthy	Type 2 diabetic	Normal pellet diet	High fat diet	Administrations (daily)	
					NO	Glibenclamide
C	+	−	+	−	—	—
CNO-10	+	−	+	−	375 *μ*g	—
HF	+	−	−	+	—	—
NO-0.1	−	+	−	+	3.75 *μ*g	—
NO-1	−	+	−	+	3.75 *μ*g	—
NO-10	−	+	−	+	375 *μ*g	—
D (negative control)	−	+	−	+	—	—
G (positive control)	−	+	−	+	—	0.6 mg/kg bw

*NO groups and glibenclamide group had administered doses in 0.5 mL of distilled water by gavage once in a day for 12 weeks.

**Table 3:** Data for body weight, fasting blood glucose, HbA1c, and lipid profiles of groups at the end of the experimental period.

Groups	Body weight g	FBG mmol/L	HbA1c %	Total-C mmol/L	HDL mmol/L	LDL mmol/L	Triglyceride mmo/L	AI	HDL% in Total-C	Triglyceride/HDL ratio
C	342.40 ± 19.73^abc^	7.99 ± 0.68^a^	4.26 ± 0.12^a^	1.74 ± 0.04^ab^	0.97 ± 0.04^ab^	0.60 ± 0.05^ab^	0.63 ± 0.09^a^	0.77 ± 0.07^ab^	55.81 ± 1.93^b^	0.65 ± 0.10^ab^
CNO-10	305.50 ± 15.42^a^	7.06 ± 0.84^a^	4.42 ± 0.04^a^	1.40 ± 0.14^a^	0.91 ± 0.05^a^	0.57 ± 0.03^a^	0.45 ± 0.04^a^	0.71 ± 0.11^ab^	58.92 ± 4.06^bc^	0.56 ± 0.05^a^
HF	375.17 ± 20.37^c^	9.95 ± 0.45^ab^	4.22 ± 0.06^a^	1.83 ± 0.06^b^	1.04 ± 0.04^abc^	0.73 ± 0.05^ab^	0.76 ± 0.10^ab^	0.77 ± 0.04^ab^	56.61 ± 1.19^b^	0.75 ± 0.10^ab^
NO-0.1	372.25 ± 9.03^c^	15.18 ± 0.90^c^	4.63 ± 0.09^a^	2.31 ± 0.08^c^	1.23 ± 0.03^d^	0.63 ± 0.02^ab^	1.16 ± 0.14^bc^	0.88 ± 0.09^bc^	52.03 ± 2.16^ab^	0.88 ± 0.13^ab^
NO-1	359.25 ± 12.13^bc^	14.82 ± 1.31^c^	4.64 ± 0.19^a^	1.96 ± 0.07^bc^	1.09 ± 0.07^bcd^	0.54 ± 0.04^a^	1.13 ± 0.14^bc^	0.89 ± 0.04^bc^	53.16 ± 1.19^ab^	0.97 ± 0.07^bc^
NO-10	387.36 ± 12.77^c^	14.35 ± 0.71^bc^	4.57 ± 0.07^a^	1.86 ± 0.08^b^	1.20 ± 0.04^d^	0.56 ± 0.02^a^	1.08 ± 0.02^bc^	0.61 ± 0.10^a^	63.27 ± 3.47^c^	0.92 ± 0.05^b^
D	298.33 ± 18.28^a^	23.98 ± 2.48^d^	5.51 ± 0.30^b^	2.86 ± 0.29^d^	1.16 ± 0.03^cd^	0.85 ± 0.20^b^	1.48 ± 0.16^c^	1.09 ± 0.05^c^	48.41 ± 1.00^a^	1.42 ± 0.17^d^
G	322.44 ± 17.61^ab^	21.34 ± 2.55^d^	5.52 ± 0.33^b^	2.27 ± 0.04^c^	1.02 ± 0.04^abc^	0.64 ± 0.04^ab^	1.32 ± 0.09^c^	1.10 ± 0.04^c^	47.68 ± 0.87^a^	1.26 ± 0.08^cd^
*P *	*P* < 0.0001	*P* < 0.0001	*P* < 0.0001	*P* < 0.0001	*P* < 0.0001	*P* < 0.05	*P* < 0.0001	*P* < 0.001	*P* < 0.0001	*P* < 0.0001

Values are mean ± SEM*.*

^a,b,c^Means with different superscript letters within a column are significantly different from each other at *P* value ( Duncan's test) which is represented at the end of same column. *P* < 0.05, significant, *P* < 0.01 and *P* < 0.001, highly significant.

FBG: fasting blood glucose; HbA1c: haemoglobin A1c; Total-C: total cholesterol; HDL: high density lipoprotein; LDL: low density lipoprotein; AI: atherogenic index.

After 2-week feeding of high fat diet, diabetes was induced in fasted animals by a single intraperitoneal injection of streptozotocin (35 mg/kg bw) dissolved in citrate buffer (pH 4.5) [11].

C: healthy control, normal diet; CNO-10: healthy control, normal diet and NO distillate at a dose of 375 *μ*g/0.5 mL of distilled water/d; HF: healthy control high fat diet;. NO-0.1: type 2 diabetic, high fat diet, and NO distillate at a dose of 3.75 *μ*g/0.5 mL of distilled water/d; NO-1: type 2 diabetic, high fat diet, and NO distillate at a dose of 37.5 *μ*g/0.5 mL of distilled water/d; NO-10: type 2 diabetic, high fat diet, and NO distillate at a dose of 375 *μ*g/0.5 mL of distilled water/d; D: type 2 diabetic, high fat diet; G: type 2 diabetic, high fat diet, and glibenclamide at a dose of 0.6 mg/kg bw/0.5 mL of distilled water/d.

**Table 4:** Values of HOMA-IR, HOMA-*β*, insulin, leptin, and liver enzymes of groups at the end of the experimental period.

Groups	Insulin pmol/L	Leptin ng/mL	HOMA-IR	HOMA-*β* %	ALP U/L	AST U/L	ALT U/L
C	40.77 ± 5.90^a^	2.61 ± 0.17^a^	2.13 ± 0.28^ab^	25.97 ± 6.19^ab^	125.00 ± 4.43^a^	130.00 ± 9.59^ab^	33.60 ± 2.71^a^
CNO-10	29.31 ± 2.22^a^	2.36 ± 0.57^a^	1.32 ± 0.34^a^	30.08 ± 7.08^b^	172.00 ± 17.51^abc^	120.00 ± 12.96^ab^	40.00 ± 6.93^ab^
HF	44.10 ± 10.07^a^	2.85 ± 0.43^a^	2.89 ± 0.69^ab^	22.83 ± 4.43^ab^	145.60 ± 9.17^ab^	118.67 ± 3.21^ab^	40.00 ± 2.83^ab^
NO-0.1	75.91 ± 15.00^a^	2.89 ± 0.24^a^	6.65 ± 1.69^b^	27.91 ± 8.53^b^	204.00 ± 13.88^abc^	134.18 ± 5.16^ab^	44.44 ± 1.94^ab^
NO-1	73.13 ± 9.51^a^	2.45 ± 0.52^a^	6.81 ± 0.90^b^	24.90 ± 6.92^ab^	202.00 ± 19.99^abc^	127.00 ± 10.90^ab^	42.80 ± 4.22^ab^
NO-10	66.60 ± 7.64^a^	2.26 ± 0.46^a^	5.64 ± 0.89^ab^	21.64 ± 2.63^ab^	218.67 ± 22.00^bc^	111.00 ± 7.04^a^	41.00 ± 2.48^ab^
D	134.59 ± 24.72^b^	4.91 ± 0.64^b^	18.45 ± 3.83^c^	7.65 ± 1.31^a^	376.00 ± 50.23^d^	146.00 ± 18.41^b^	52.00 ± 3.43^b^
G	49.45 ± 2.15^a^	4.71 ± 0.96^b^	7.24 ± 1.83^b^	8.20 ± 2.01^a^	250.86 ± 29.13^c^	118.67 ± 4.00^ab^	51.00 ± 3.68^b^
*P *	*P* < 0.0001	*P* < 0.01	*P* < 0.0001	*P* < 0.05	*P* < 0.0001	*P* < 0.05	*P* < 0.05

Values are mean ± SEM.

^a,b,c^Means with different superscript letters within a column are significantly different from each other at *P* value (Duncan's test) which is represented at the end of same column. *P* < 0.05, significant, *P* < 0.01 and *P* < 0.001, highly significant.

HOMA-IR: homeostasis model assessment of insulin resistance (HOMA-IR = [fasting insulin (μIU/mL) × (fasting glucose (mmol/L)]/22.5); HOMA-*β*: homeostasis model assessment of beta cell function (HOMA-*β*(%)= (20 × fasting insulin (*μ*U/mL)/{fasting glucose (mmol/L) − 3.5}).ALP: alkaline phosphatase, AST: aspartate transaminase, ALT: alanine transaminase.

After 2-week feeding of high fat diet, diabetes was induced in fasted animals by a single intraperitoneal injection of streptozotocin (35 mg/kg bw) dissolved in citrate buffer (pH 4.5) [11].

C: healthy control, normal diet; CNO-10: healthy control, normal diet, and NO distillate at a dose of 375 *μ*g/0.5 mL of distilled water/d; HF: healthy control high fat diet; NO-0.1: type 2 diabetic, high fat diet, and NO distillate at a dose of 3.75 *μ*g/0.5 mL of distilled water/d; NO-1: type 2 diabetic, high fat diet, and NO distillate at a dose of 37.5 *μ*g/0.5 mL of distilled water/d; NO-10: type 2 diabetic, high fat diet, and NO distillate at a dose of 375 *μ*g/0.5 mL of distilled water/d; D: type 2 diabetic, high fat diet; G: Type 2 diabetic, high fat diet, and glibenclamide at a dose of 0.6 mg/kg bw/0.5 mL of distilled water/d.

**Table 5:** Primer sequences of PPAR-*α*, PPAR-*β*, PPAR-*γ*, *β*-Actin.

	Primer sequences	PCR product size-bp	At (°C)
PPAR-*α*	5′c ccaatggttgctgattaca-3 5′ggacgcaggctctactttga-3	580	56
PPAR-*β*	5′cccaatggttgctgattaca-3 5′ggacgcaggctctactttga-3	202	56
PPAR-*γ*	5′tgcggactaccagtacttaggg-3 5′ggaagctggagagagggtgt-3	210	59
*β*-Actin	5′ccctcatagatgggcacagt-3	4477	50
	5′gtagccatccaggctgtgtt-3		
PCR Protocol	92°C 1 min 60°C 1 min 72°C 1 min		
